# Key residues of *Bacillus thuringiensis* Cry2Ab for oligomerization and pore-formation activity

**DOI:** 10.1186/s13568-021-01270-0

**Published:** 2021-07-31

**Authors:** Zhi-Zhen Pan, Lian Xu, Bo Liu, Qing-Xi Chen, Yu-Jing Zhu

**Affiliations:** 1 Agricultural Bio-Resources Research Institute, Fujian Academy of Agricultural Sciences, Fuzhou, 350003 China; 2 School of Life Sciences, Xiamen University, Xiamen, 361005 China

**Keywords:** Cry2Ab, Oligomerization, Insecticidal activity, Pore-forming activity

## Abstract

As a pore-forming toxin, activation, oligomerization and pore-formation were both required for the mode of action of Cry toxins. Previous results revealed that the helices α4–α5 of Domain I were involved in the oligomerization of Cry2Ab, however, the key residues for Cry2Ab aggregation remained ambiguous. In present studies, we built 20 Cry2Ab alanine mutants site-directed in the helices α4–α5 of Domain I and demonstrated that mutants N151A, T152A, F157A, L183A, L185A and I188A could reduce the assembly of the 250 kDa oligomers, suggesting that these mutation residues might be essential for Cry2Ab oligomerization. As expected, all of these variants showed lower insecticidal activity against *P. xylostella*. Furthermore, we found that the pore-forming activities of these mutants also decreased when compared to wild-type Cry2Ab. Taken together, our data identified key residues for Cry2Ab oligomerization and emphasized that oligomerization was closely related to the insecticidal activity and pore-forming activity of Cry2Ab.

**Supplementary Information:**

The online version contains supplementary material available at 10.1186/s13568-021-01270-0.

## Introduction

Cry toxin, an insecticidal crystal toxin derived from *Bacillus thuringiensis* (Bt), is widely applied as a bio-insecticides to control agricultural pests all over the world (Bravo et al. 2011; Schnepf et al. 1998). In general, Cry toxins are produced in an inactive form called protoxin and proteolytic activated by target insect midgut protease. Activated Cry toxins interacted with the midgut receptors in target insects, self-aggregated to form pre-pore oligomeric structures and inserted into the cell membrane to form pores, resulting in the destruction of the midgut epithelium and the death of insects (Pardo-López et al. 2013; Bravo et al. 2007; Vachon et al. 2012).

At present, most researches have been devoted into “interaction of Cry toxins with midgut receptors” which is an essential step for the insecticidal mechanism of Cry toxins (Pigott and Ellar 2007; Bravo et al. 2013). As a result, a number of insect midgut proteins such as cadherin, aminopeptidase-N (APN), alkaline phosphatase (ALP) and ATP-binding cassette transporter subfamily C member 2 (ABCC2) have been proposed as functional receptors and mediated the insecticidal activities of Cry toxins (Pigott and Ellar 2007; Zhou et al. 2017; Park et al. 2009; Guo et al. 2015; Chen et al. 2017). It was suggested that the mutation or down-regulation of Cry receptor genes were tightly linked with high level of Cry resistance in diverse insects (Ferré and Van Rie 2002; Baxter et al. 2011). It was also reported that the improper processing of Cry toxins was associated with Cry resistance in lepidopteran insects (Liu et al. 2014; Xia et al. 2016). Those studies made a closer look on the mechanism of Cry toxins and provided new strategies for agricultural pest control.

As a pore-forming toxin, Cry toxins kill insects by forming pore on their midgut cells. Assembly of pre-pore oligomers was required for pore-forming processing of Cry toxins (Jiménez-Juárez et al. 2013; Vachon et al. 2002). Domain I, comprised by seven-helix bundle, was widely reported involving in the assembly of pre-pore structure and membrane channel formation (Pardo-López et al. 2013; Vachon et al. 2012). It was proposed that a binding step of cadherin receptors and proteolytic removal of helix α-1 were necessary for oligomerization of Cry1A toxin (Soberón et al. 2007). Helix α-3 was found to participate in toxin oligomerization as the mutation on this region affected oligomerization, as well as toxicity of Cry toxin (Vachon et al. 2002; Muñoz-Garay et al. 2009). Recently, Pacheco et al. reported that an intramolecular salt bridge in helix α-3 was essential for the stability and oligomerization of Cry4Ba toxins (Pacheco et al. 2017). The helices α-4 and α-5 had been wildly reported as a pore-forming region, as they were the only helices capable of adopting a transmembrane orientation (Girard et al. 2008; Torres et al. 2008). An umbrella model was further put forward in which the helices α-4 and α-5 inserted into the membrane to form pores while other α‐helices covered the surface of membrane (Pardo-López et al. 2013).

Our previous study suggested that the exposure of helices α4–α5 was required for oligomerization and insecticidal activity of Cry2Ab (Xu et al. 2018). However, the key residues involved in the oligomerization activity of Cry2Ab were still unknown. In the present study, we sought to determine the key residues of Cry2Ab for oligomerization activity by constructing 20 alanine mutants site directed on helices α4–α5. It revealed that residues N151, T152, F157, L183, L185 and I188 are involved in Cry2Ab oligomerization. The Cry2Ab mutants with lower activities of oligomerization not only reduced the insecticidal activities against *Plutella xylostella*, but also weakened the pore-forming activities on liposome. Our data firstly identified key residues for Cry2Ab oligomerization and highlighted that oligomerization was linked with the insecticidal activity and pore-forming activity of Cry2Ab.

## Materials and methods

### Insects

A laboratory population of *P. xylostella* larvae was purchased from Henan Jiyuan Baiyun Industry Co., Ltd, China. *P. xylostella* larvae were fed with an artificial diet and maintained under environmental conditions of 27 ± 2 °C, 70% of humidity, and photoperiod of 14:10 h (light/dark).

### Construction of Cry2Ab variants

Twenty Cry2Ab mutants site-directed on the helices α4–α5 of Domain I were built by replacing residues V150, N151, T152, M153, Q154, Q155, L156, F157, L158, N159, R160, N182, L183, H184, L185, S186, F187, I188, R189, D190 with alanine. The Cry2Ab mutants were obtained by overlapping-extension PCR using wild-type *cry2Ab* DNA fragment as template (Xu et al. 2018). Primers used for generation of site-directed mutagenesis were listed in Additional file 1: Table S1. The mutated *cry2Ab* fragments were further digested by the restriction enzymes BglII and EcoRI, and then ligated into plasmid pET30a. Recombined plasmids were transformed into *Escherichia coli* BL21 (DE3) cells and the positive clones were verified by PCR, restriction enzyme digestion and DNA sequencing (Additional file 1: Figs. S1–S3).

### Expression of Cry2Ab variants

The production and purification of wild-type and mutant Cry2Ab toxins were performed as previously described (Pan et al. 2014). *E. coli* BL21 (DE3) cells harboring pET30a-*cry2Ab* were grown in LB medium containing 35 µg/mL kanamycin. The expression of wild-type and mutants Cry2Ab toxins were induced overnight at 25 °C with 0.5 mM isopropyl-B-d-thiogalactopyranoside (IPTG) after OD_600 nm_ reached 0.4–0.6. Cells were pelleted at 6000 g under 4 °C and resuspended with Tris-HCl buffer (25 mM Tris-HCl, 300 mM NaCl, 25 mM imidazole, pH 7.0) and then lysed by ultrasonication. The soluble Cry2Ab toxins were purified by a Ni-IDA Prepacked Column (Sangon, China) according to the instruction manual. Purified Cry2Ab were detected by 10% SDS-PAGE and western blotting using an anti-Cry2Ab antibody. Protein concentration of Cry2Ab toxins were determined using a BCA Protein Assay Kit (Beyotime, China).

### Oligomerization assay

Cry2Ab protoxin was incubated with *P. xylostella* midgut juice (PxMJ) with a mass ratio of 20:1 (m: m) at 30 °C for 60 min and exchanged into sodium carbonate buffer (50 mM, pH 9.5) using a PD-10 desalting column. The brush border membrane vesicles from *P. xylostella* midguts were prepared as Wolfersberger et al. reported (1987). For oligomeric formation assays, 20 µg of Cry2Ab activated-toxin was incubated at 30 °C overnight in the present of PxBBMV (10 µg). The oligomeric formation of Cry2Ab was detected by 8% SDS-PAGE with Coomassie staining (Xu et al. 2018). The ratio of oligomerization percentage was calculated by Image J software to evaluate the percentage of oligomer (oligomer/monomer × 100%).

### Bioassay

Bioassays of wild-type and mutants Cry2Ab toxins against second instar larvae of *P. xylostella* were estimated according to Pan et al. (2014). A 2 mL artificial diet was added to 6-well polystyrene plates (Sangon, China) and air-dried. Five or six concentrations of Cry2Ab toxins from 0.032 to 20 µg/cm^2^ were set up. Insects tested with sodium carbonate buffer (50 mM, pH 9.5) were served as the negative control. Thirty second instar larvae of *P. xylostella* were used for each concentration assay and three independent replicates were performed. Observations were recorded at 48 h, and the median lethal concentration (LC_50_) value was analyzed by SPSS 17.0 (Statistical Product and Service Solutions) using PROBIT analysis (Finney 1971). The relative potency (%) had been normalized to the LC_50_ value of wild-type Cry2Ab.

### Liposome leakage assay

Liposome preparation was performed as described by Ding et al. (2016). Phosphatidylcholine, phosphatidylethanolamine and cholesterol were dissolved in chloroform and mixed in a 4:4:2 proportion (molar mass). The mixed lipids were put in a glass vial and evaporated under a stream of nitrogen to form lipid film. The lipid film was then shaking with SUV-1 buffer (20 mM HEPES, 50 mM NaCl, 3 mM calcein, pH 7.5) at room temperature for 2 h. Liposomes were generated by extrusion of the hydrated lipids through a 100-nm polycarbonate filter (Whatman) 35 times using a Mini-Extruder device (Avanti Polar Lipids Inc). Calcein, outside the liposome, were removed by exchanging the liposome into SUV-2 buffer (20 mM HEPES, 50 mM NaCl, pH 7.5) using a Sephadex G-50 column. Liposomes were stored at 4 °C and used within 48 h.

For liposome leakage assay, the liposome encapsulated calcein was diluted to 200 µM in SUV-2 buffer supplemented with 3 µM MnCl_2_. The released calcein could be quenched by MnCl_2_ in the solution. The excitation and emission wavelengths were set as 490 nm and 520 nm, respectively, to examine the fluorescence of calcein. 480 µL of liposome was added to the cuvette and the emission fluorescence was readed as F_t0_. 20 µL of activated-Cry2Ab (10 µg) was then added to and the emission fluorescence was continuously recorded as Ft at 10 s intervals. After 10 min, 20 µL of 10% Triton X-100 was added to achieve complete release of calcein and the fluorescence records were defined as F_t100_. The percentage of liposome leakage at each time point is defined as: leakage (t) (%) = (F_t_ − F_t0_) × 100/(F_t100_ − F_t0_).

## Results

### Construction of Cry2Ab mutants sited-directed on helices α4–α5

**Fig. 1:**
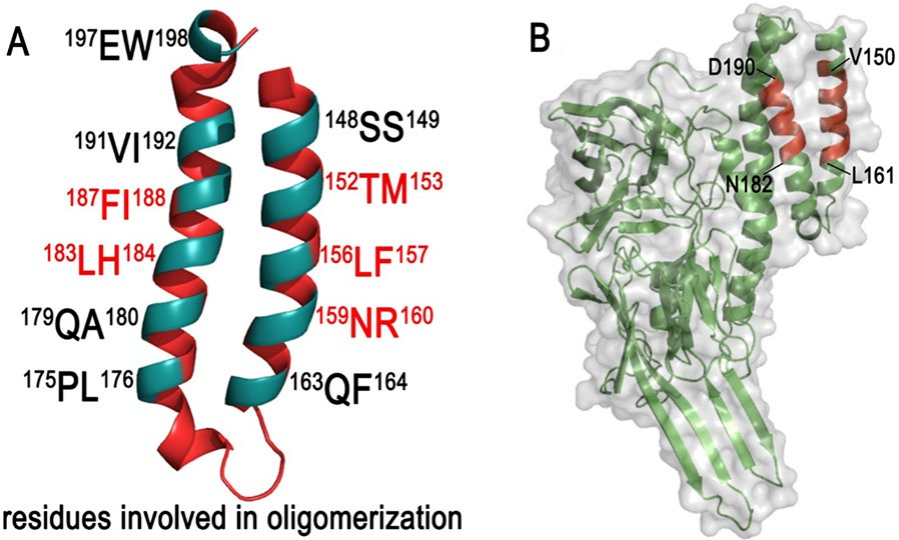
The design and construction of alanine mutants of Cry2Ab site-directed on the helices α4–α5. **A** Helices α4–α5 in Domain I of Cry2Ab. **B** The 3D structure of Cry2Ab activated-toxin, proteolysis of Cry2Ab removed the first three α-helices (helices α1–α3) and exposed the helices α4–α5 in Domain I. The region V^150^-R^160^ in helix α4 and N^182^-D^190^ in helix α5 were speculated involved in Cry2Ab oligomerization

Our previous studies demonstrated that the helices α4–α5 in Domain I was involved in oligomerization of Cry2Ab since some Cry2Ab mutants (TM152153AA, LF156157AA, NR159160AA, LH183184AA, FI187188AA) failed to assemble 250 kDa oligomers (Fig. 1A). Those results suggested that the active regions for Cry2Ab oligomerization might limited to V^150^-R^160^ in helix α-4 and N^182^-D^190^ in helix α-5 (Fig. 1B). To further authenticate the key residues for Cry2Ab oligomerization, 11 Cry2Ab mutants site-directed on helix α-4 (V150A, N151A, T152A, M153A, Q154A, Q155A, L156A, F157A, L158A, N159A, R160A) and 9 Cry2Ab mutants site-directed on helix α-5(N182A, L183A, H184A, L185A, S186A, F187A, I188A, R189A, D190A)were constructed using *Escherichia coli* Bl21(DE3) expression system.

### Production and identification of Cry2Ab mutants

**Fig. 2:**
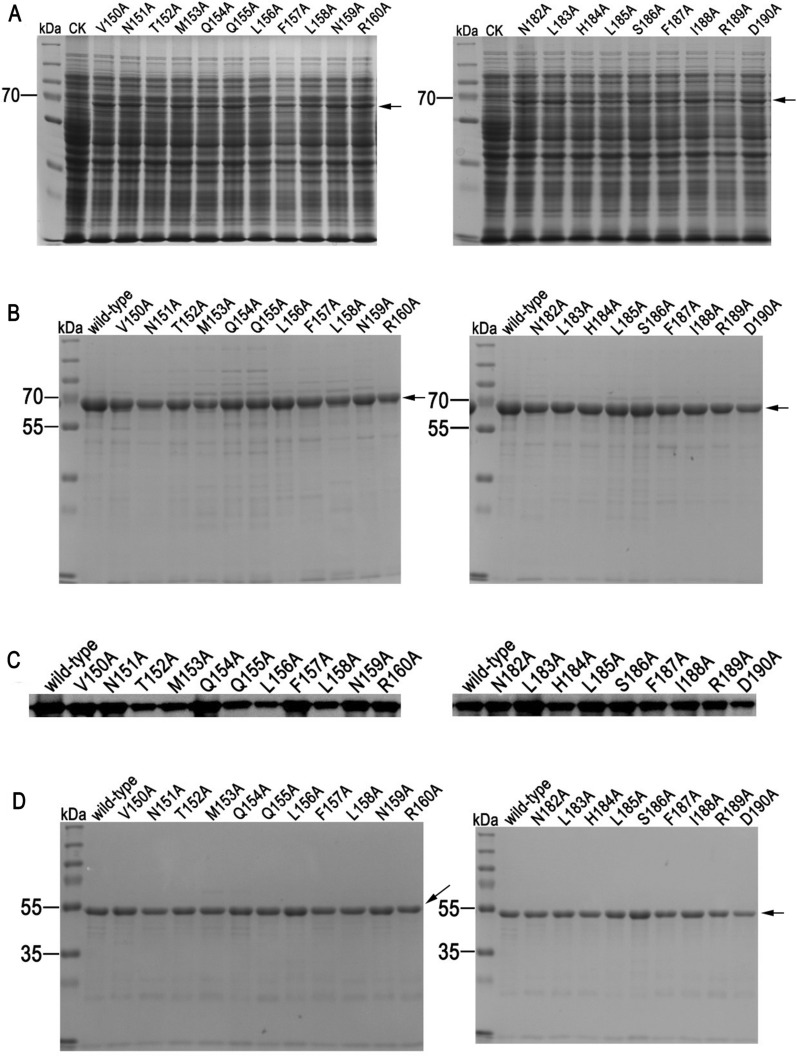
Expression, purification and identification of Cry2Ab mutants. **A** Induced expression of Cry2Ab variants. **B** The purity of Cry2Ab mutants detected by SDS-PAGE followed by Coomassie blue staining. **C** Identification of Cry2Ab mutants using an anti-Cry2Ab antibody. **D** The proteolytic activation of Cry2Ab mutants by PxMJ

The expression of Cry2Ab variants were performed under the induction of 0.5mM IPTG. SDS-PAGE revealed that all Cry2Ab mutants could be induced to express with a molecular weight of 65 kDa (Fig. 2A). Those Cry2Ab mutants further purified by a Ni-IDA prepacked column (Fig. 2B) and could be detected by an anti-Cry2Ab antibody (Fig. 2C). Furthermore, proteolysis assay indicated that all Cry2Ab variants could be processed into 50 kDa activated-toxins by PxMJ, which were similar to wild-type Cry2Ab (Fig. 2D). These results revealed that mutations in the helices α4–α5 did not cause a major structural disturbance in Cry2Ab.

### Authentication of key residues for Cry2Ab oligomerization

**Fig. 3:**
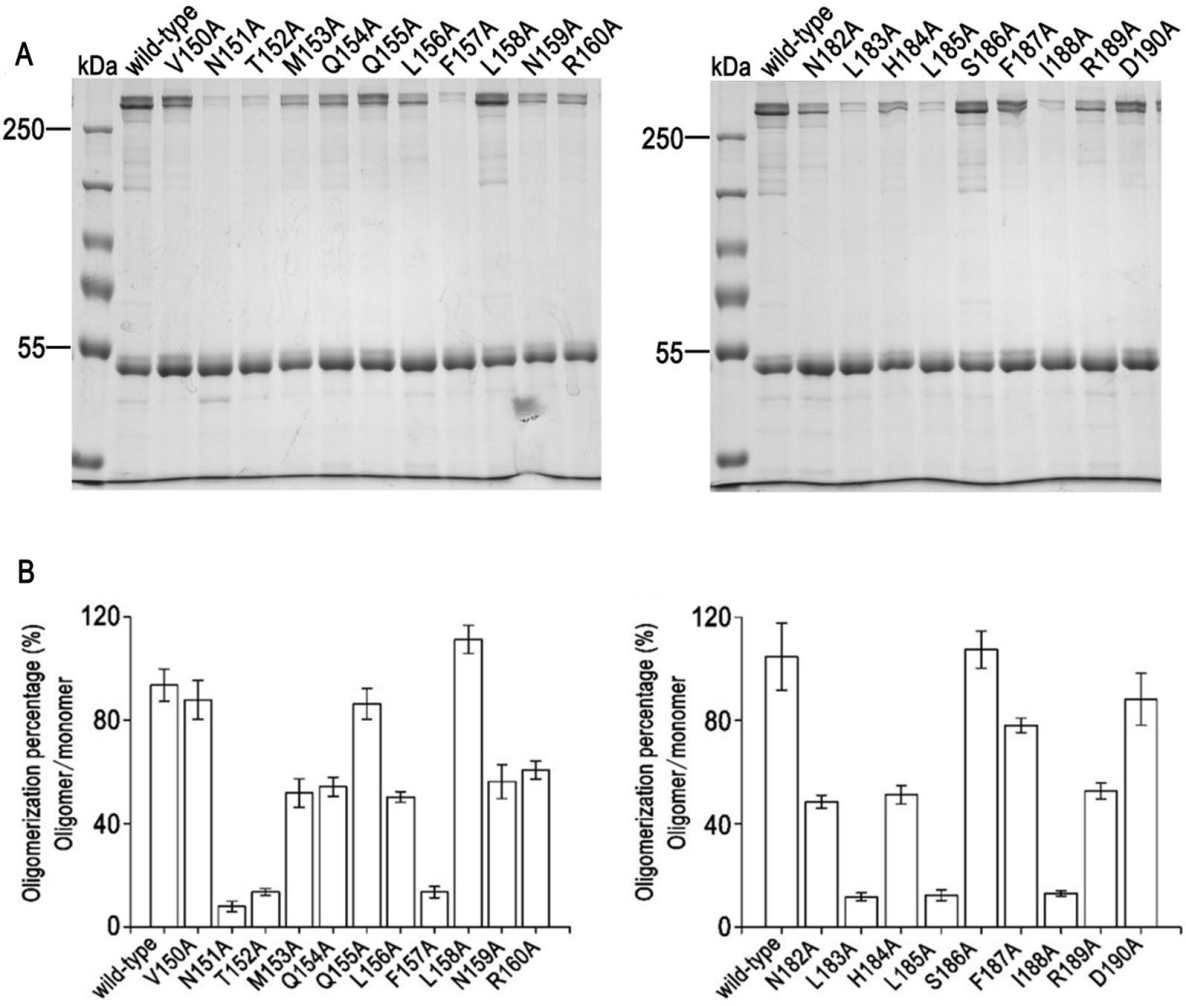
Oligomerization assay of Cry2Ab mutants. **A** Assemble of Cry2Ab oligomers. **B** Oligomerization percentage of Cry2Ab was calculated by image J. The data were presented with the mean value ± standard deviations from triplicate biological experiments

The assembly of 250 kDa oligomer of Cry2Ab variants were evaluated by 8% SDS-PAGE (Fig. 3A). Wild-type Cry2Ab could form 250 kDa oligomers which was similar to our previous reports (Xu et al. 2018). By contrast, six Cry2Ab mutants (N151A, T152A, F157A in helix α-4 and L183A, L185A, I188A in helix α-5) barely formed the 250 kDa oligomers (defined as non-oligomerization group). Eight Cry2Ab mutants (M153A, Q154A, L156A, N159A, R160A in helix α-4 and N182A, H184A, R189A in helix α-5) reduced the assembly of 250 kDa oligomers (defined as reduced oligomerization group). The remaining Cry2Ab variants (V150A, Q155A, L158A in helix α-4 and S186A, F187A, D190A in helix α-5) could aggregate and form 250 kDa pre-pore structure (defined as normal oligomerization group) which were similar to wild-type Cry2Ab.

To further assess the oligomers formation of Cry2Ab, we employed Image J software to evaluate the percentage of Cry2Ab oligomer (oligomer/monomer × 100%). As shown in Fig. 3B, the proportion of oligomer and monomer in normal oligomerization group was about 80–110%, which was similar to wild-type Cry2Ab (the percentage was about 95%). However, this value went down to 40–60% in reduced oligomerization group and were less than 20% in non-aggregation group. These results suggested that residues N151, T152, F157, L183, L185, I188 might serve as key residues for Cry2Ab oligomerization.

### Oligomerization is associated with insecticidal activity of Cry2Ab

We then evaluated the insecticidal activities of non-oligomerization Cry2Ab mutants (N151A, T152A, F157A, L183A, L185A and I188A) against second instar larvae of *P. xylostella*, with wild-type Cry2Ab, V150A and S186A (two Cry2Ab mutants in normal oligomerization group) as positive control. As shown in Table 1, the LC_50_ values of V150A and S186A was 1.978 and 1.432 µg/cm^2^, respectively, which were close to that of wild-type Cry2Ab (1.458 µg/cm^2^). However, the LC_50_ values of N151A, T152A, F157A, L183A, L185A and I188A were 5.097, 4.232, 3.234, 3.083, 3.579 and 3.545 µg/cm^2^, respectively, all of which were higher than that of wild-type Cry2Ab. These results indicated that oligomerization was associated with the insecticidal activity of Cry2Ab.

**Table 1:** The median lethal concentration (LC_50_) of wild-type Cry2Ab and its variants against second‐instar larvae of *Plutella xylostella*

Cry2Ab	LC_50_ (µg/cm^2^)	95% confidence level (µg/cm^2^)	Relatively LC_50_ (%)
Wild-type	1.458	0.738–2.445	100
V150A	1.978	0.946–2.687	73.7
S186A	1.432	0.793–2.487	102
N151A	5.097	3.874–10.343	28.6
T152A	4.232	3.045–8.265	34.5
F157A	3.234	2.904–8.249	45.1
L183A	3.083	1.568–7.675	47.3
L185A	3.579	2.511–9.735	40.7
I188A	3.545	2.987–10.734	41.1

### Oligomerization is linked with pore-forming activity of Cry2Ab

**Fig. 4:**
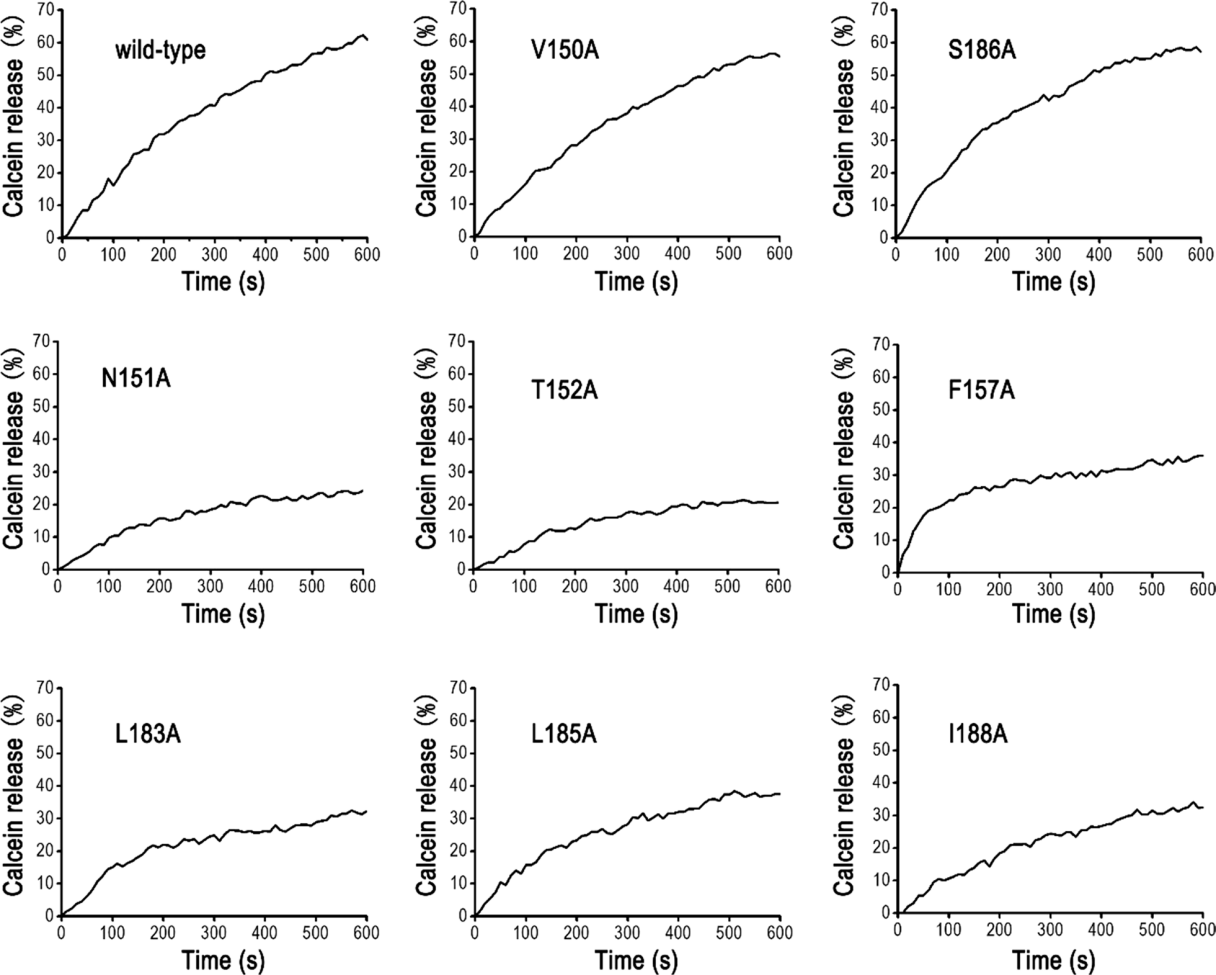
Liposome leakage assay detected pore-forming activity of Cry2Ab mutants

The pore-forming activities of non-oligomerization Cry2Ab mutants (N151A, T152A, F157A, L183A, L185A and I188A) were further assessed by time course of liposome leakage assay. As shown in Fig. 4, wild-type Cry2Ab activated-toxin could make pore on liposome and led to high leakage of calcein into solution, which was the same with V150A and S186A variants. The maximum calcein release percentage caused by wild-type Cry2Ab, V150A and S186A activated-toxin were 60.95%, 55.87%, and 58.73%, respectively. However, the leakage of calcein caused by N151A, T152A, F157A, L183A, L185A and I188A activated-toxins were only 24.33%, 20.72%, 35.98%, 32.36%, 37.53 and 32.53%, all of which were lower than wild-type Cry2Ab. These data demonstrated that non-oligomerization mutants could weaken the pore-forming activities on liposome. Taken together, our data indicated that oligomerization was linked with the pore-forming activity of Cry2Ab.

## Discussion

The helices α-4 and α-5 of Cry1A had been wildly reported as a pore-forming region (Girard et al. 2008; Torres et al. 2008). In other Cry toxin, helices α-4 and α-5 also reported in the oligomerization activity. Kanintronkul et al. reported that N166 and Y170 in helices α4–α5 was involved in the aggregation of Cry4B (Kanintronkul et al. 2003). Pornwiroon et al. reported that Y202 in helices α4 was responded to the oligomerization activity of Cry4A (Pornwiroon et al. 2004). In our previous study also suggested that helices α4–α5 participate in Cry2Ab self-aggregation (Xu et al. 2018). In this study, we further constructed 20 alanine mutants site directed on helices α4–α5 and showed that residues N151, T152, F157, L183, L185 and I188 may be key residues for Cry2Ab oligomerization and insecticidal activity.

The pore-formation of Cry toxins on the membrane was one of the less characterized steps and was indispensable to fully understand the mechanism action of Cry toxins (Muñoz-Garay et al. 2006). Zavala et al. demonstrated that only Domain I inserted into the liposome while Domain II and III remained in the surface of membrane (Zavala et al. 2011). This result was consistence with the umbrella model of toxin insertion. It also revealed that residues V171 and T122 involved in the insertion of Cry1A toxin into members by fluorescent studies. Angsuthanasombat et al. revealed that residue Arg-136 participated in the membrane channel formation of Cry4Ba (Angsuthanasombat et al. 2001). It was also reported that residue N183 located in the middle of helix α-5 was crucial for the insecticidal activity and pore-forming activity of Cry4Ba (Likitvivatanavong et al. 2006). Similarly, our data showed that mutations on helice α-4 and α-5 (N151A, T152A, F157A, L183A, L185A and I188A) also blocked the pore-formation of Cry2Ab on liposome.

It was worth mentioning that Cry2Ab mutants which failed to assemble 250 kDa oligomers, not only disrupted the insecticidal activity against *P. xylostella*, but also lost pore-forming activity of liposome. The results suggested that oligomerization and pore-formation was closely related in Cry2Ab. This mode of action was quite different from that of Cry1A toxin. For example, helices α-3 and α-6 of Cry1A was reported involved in oligomerization while helices α-4 and α-5 participated in pore-formation (Jiménez-Juárez et al. 2013; Lin et al. 2014). Furthermore, in Cry1A toxin, it was reported that toxins oligomerization required a binding step to cadherin, which promoted to cleavage of helix α-1 (Soberón et al. 2007). However, our previous data revealed that Cry2Ab could oligomerize in vitro after proteolysis by PxMJ in the absence of the cadherin (Xu et al. 2018). This diversity suggested that the mode of action of Cry2Ab might different from Cry1A toxin in some details and more researches were needed to clarify the elaborate difference. In conclusion, our study comprehensively identified key residues for Cry2Ab for the first time and demonstrated that non-oligomerization mutants affected the insecticidal activity as well as the pore-forming activity of Cry2Ab. It highlighted that oligomerization was closely related to insecticidal activity and pore-forming activity of Cry2Ab, which could make a closer look on the mode of action of Cry2Ab toxins. Further studies will focus on the saturated mutation of these key residues to construct Cry2Ab variants with enhanced oligomerization activities as well as higher insecticidal performance.

## Supplementary Information

**Additional file 1: TableS1.** Primer sequences used for the generation of mutants Cry2Ab. **Figure S1. **(A) Amplification of front and rear *cry2Ab* helix-α4 mutant DNA by PCR; (B) Amplification of front and rear *cry2Ab* helix-α5 mutant DNA by PCR; (C) Amplification of full-length DNA fragment of *cry2Ab* mutant by overlap extension PCR. **Figure S2. **Verification of recombinant plasmid by colony PCR. (A) Cry2Ab mutants in helix-α4; (B) Cry2Ab mutants in helix-α5. **Figure S3. **Verification of recombinant plasmid by restriction enzyme digestion. (A) Cry2Ab mutants in helix-α4; (B) Cry2Ab mutants in helix-α5.

## Data Availability

The supplementary materials are available online.
