# Supplementary material for: Key residues of Bacillus thuringiensis Cry2Ab for oligomerization and pore-formation activity
Source: AMB Express. 2021 Jul 31;11:112. doi: 10.1186/s13568-021-01270-0 (PMC8325727; doi:10.1186/s13568-021-01270-0)
Supplement: Supplementary file 1 — Additional file 1: TableS1. Primer sequences used for the generation of mutants Cry2Ab. Figure S1. (A) Amplification of front and rear cry2Ab helix-α4 mutant DNA by PCR; (B) Amplification of front and rear cry2Ab helix-α5 mutant DNA by PCR; (C) Amplification of full-length DNA fragment of cry2Ab mutant by overlap extension PCR. Figure S2. Verification of recombinant plasmid by colony PCR. (A) Cry2Ab mutants in helix-α4; (B) Cry2Ab mutants in helix-α5. Figure S3. Verification of recombinant plasmid by restriction enzyme digestion. (A) Cry2Ab mutants in helix-α4; (B) Cry2Ab mutants in helix-α5. [file 13568_2021_1270_MOESM1_ESM.docx]

**Additional file 1**

**Key residues of *Bacillus thuringiensis* Cry2Ab for oligomerization and pore-formation activity**

Zhi-Zhen Pan ^1^, Lian Xu ^2^, Bo Liu ^1^, Qing-Xi Chen ^2^ and Yu-Jing Zhu ^1^*

^1^ Agricultural Bio-Resources Research Institute, Fujian Academy of Agricultural Sciences, Fuzhou 350003, China.

^2^ School of Life Sciences, Xiamen University, Xiamen 361005, China.

* Corresponding authors: Tel/Fax: (+86)059183723032. E-mail: [zyjingfz@163.com](mailto:zyjingfz@163.com) (Yu-Jing Zhu)

**Table S1.** Primer sequences used for the generation of mutants Cry2Ab.

| primer | primer sequence (5’-3’) |
| --- | --- |
| Cry2Ab F | GCAGATCTATGAATAGTGTATTG |
| Cry2Ab R | CGGAATTCTTAATAAAGTGGTG |
| T7 F | TAATACGACTCACTATAGGG |
| T7 R | TGCTAGTTATTGCTCAGCGG |
| 150 F | TCAATAACTTCTTCAgcaAATACAATGCAACAA |
| 150 R | TTGTTGCATTGTATTtgcTGAAGAAGTTATTGA |
| 151 F | ATAACTTCTTCAGTTgcaACAATGCAACAATTA |
| 151 R | TAATTGTTGCATTGTtgcAACTGAAGAAGTTAT |
| 152 F | ACTTCTTCAGTTAATgcaATGCAACAATTATTT |
| 152 R | AAATAATTGTTGCATtgcATTAACTGAAGAAGT |
| 153 F | TCTTCAGTTAATACAgcaCAACAATTATTTCTA |
| 153 R | TAGAAATAATTGTTGtgcTGTATTAACTGAAGA |
| 154 F | TCAGTTAATACAATGgcaCAATTATTTCTAAAT |
| 154 R | ATTTAGAAATAATTGtgcCATTGTATTAACTGA |
| 155 F | GTTAATACAATGCAAgcaTTATTTCTAAATAGA |
| 155 R | TCTATTTAGAAATAAtgcTTGCATTGTATTAAC |
| 156 F | AATACAATGCAACAAgcaTTTCTAAATAGATTA |
| 156 R | TAATCTATTTAGAAAtgcTTGTTGCATTGTATT |
| 157 F | ACAATGCAACAATTAgcaCTAAATAGATTACCC |
| 157 R | GGGTAATCTATTTAGtgcTAATTGTTGCATTGT |
| 158 F | ATGCAACAATTATTTgcaAATAGATTACCCCAG |
| 158 R | CTGGGGTAATCTATTtgcAAATAATTGTTGCAT |
| 159 F | CAACAATTATTTCTAgcaAGATTACCCCAGTTC |
| 159 R | GAACTGGGGTAATCTtgcTAGAAATAATTGTTG |
| 160 F | ACAATTATTTCTAAATgcaTTACCCCAGTTCCAG |
| 160 R | CTGGAACTGGGGTAAtgcATTTAGAAATAATTGT |
| 182 F | TTTGCACAGGCAGCCgcaTTACATCTTTCTTTT |
| 182 R | AAAAGAAAGATGTAAtgcGGCTGCCTGTGCAAA |
| 183 F | GCACAGGCAGCCAATgcaCATCTTTCTTTTATT |
| 183 R | AATAAAAGAAAGATGtgcATTGGCTGCCTGTGC |
| 184 F | CAGGCAGCCAATTTAgcaCTTTCTTTTATTAGA |
| 184 R | TCTAATAAAAGAAAGtgcTAAATTGGCTGCCTG |
| 185 F | GCAGCCAATTTACATgcaTCTTTTATTAGAGAT |
| 185 R | TCTATTTAGAAATAAtgcTTGCATTGTATTAAC |
| 186 F | GCCAATTTACATCTTgcaTTTATTAGAGATGTT |
| 186 R | AACATCTCTAATAAAtgcAAGATGTAAATTGGC |
| 187 F | AATTTACATCTTTCTgcaATTAGAGATGTTATT |
| 187 R | AATAACATCTCTAATtgcAGAAAGATGTAAATT |
| 188 F | TTACATCTTTCTTTTgcaAGAGATGTTATTCTA |
| 188 R | TAGAATAACATCTCTtgcAAAAGAAAGATGTAA |
| 189 F | CATCTTTCTTTTATTgcaGATGTTATTCTAAAT |
| 189 R | ATTTAGAATAACATCtgcAATAAAAGAAAGATG |
| 190 F | CTTTCTTTTATTAGAgcaGTTATTCTAAATGCA |
| 190 R | TGCATTTAGAATAACtgcTCTAATAAAAGAAAG |

Enzyme sites *Bgl* II and *Eco*R I were underlined. The mutant and replacement nucleotide sequences were shown in lower case text.

**Figure. S1.**


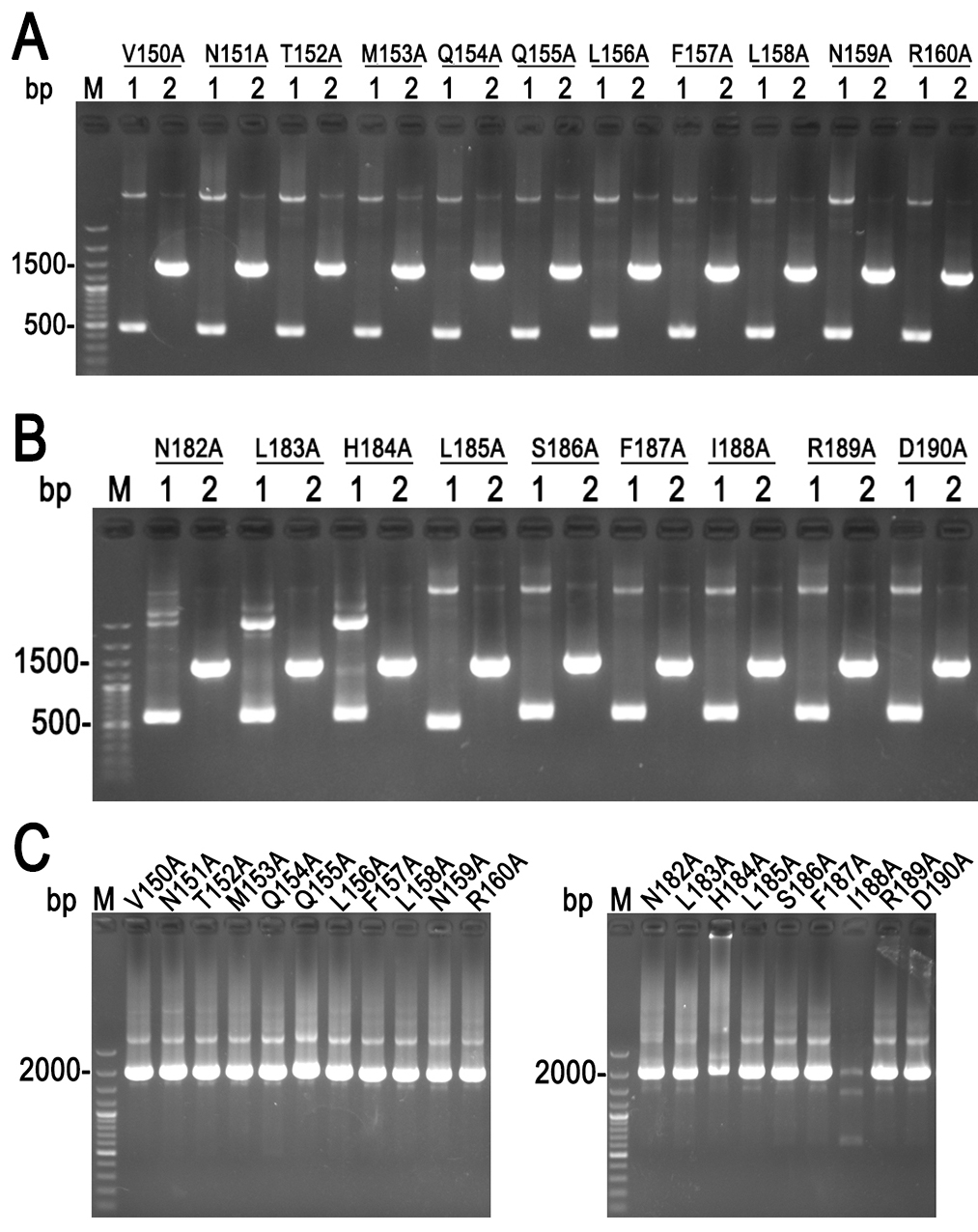


**Figure. S1.** (A) Amplification of front and rear *cry2Ab* α-4 helix mutant DNA by PCR; (B) Amplification of front and rear *cry2Ab* α-5 helix mutant DNA by PCR; (C) Amplification of full-length DNA fragment of *cry2Ab* mutant by overlap extension PCR.

**Figure. S2.**


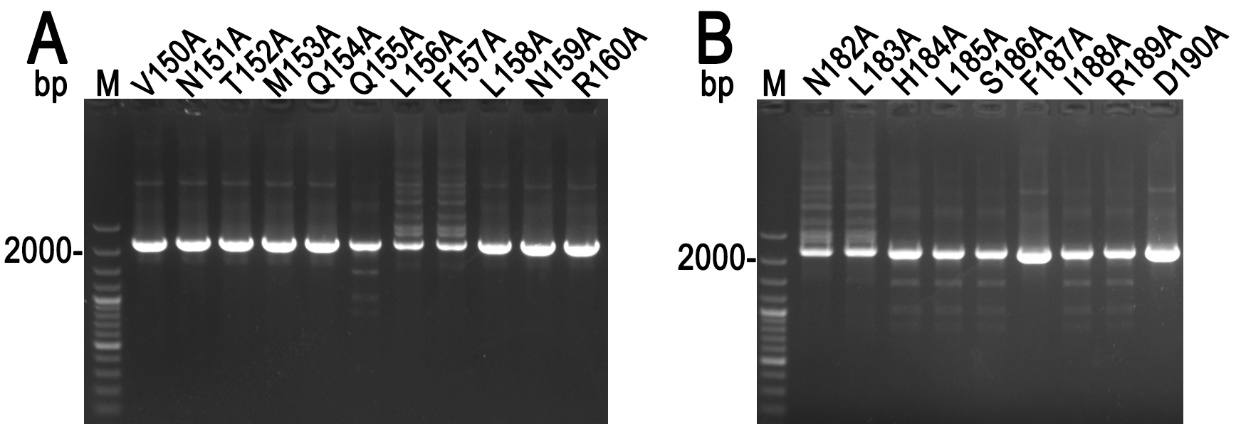


**Figure. S2.** Verification of recombinant plasmid by colony PCR. (A) Cry2Ab mutants in helix-α4; (B) Cry2Ab mutants in helix-α5.

**Figure. S3.**


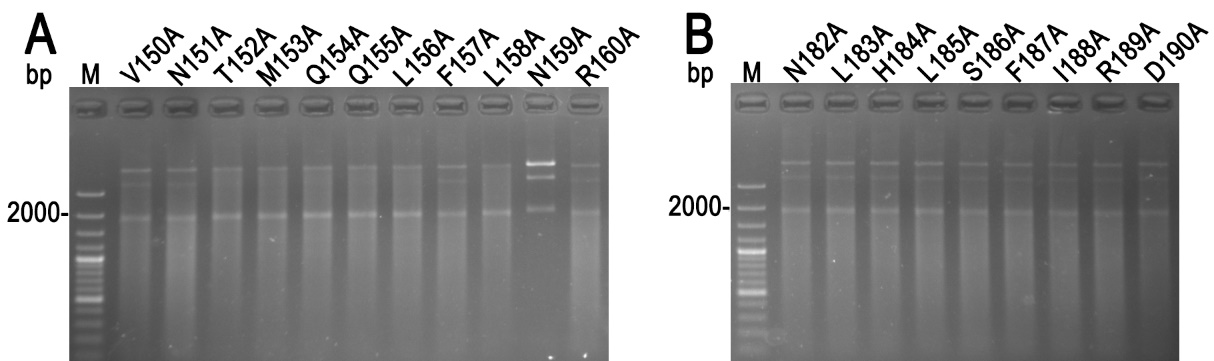


**Figure. S3.** Verification of recombinant plasmid by restriction enzyme digestion. (A) Cry2Ab mutants in helix-α4; (B) Cry2Ab mutants in helix-α5.
